# An updated comparison of microarray and RNA-seq for concentration response transcriptomic study: case studies with two cannabinoids, cannabichromene and cannabinol

**DOI:** 10.1186/s12864-025-11548-3

**Published:** 2025-04-23

**Authors:** Xiugong Gao, Miranda R. Yourick, Kayla Campasino, Yang Zhao, Estatira Sepehr, Cory Vaught, Robert L. Sprando, Jeffrey J. Yourick

**Affiliations:** Division of Toxicology, Office of Chemistry and Toxicology (OCT), Office of Laboratory Operations and Applied Science (OLOAS), Human Foods Program (HFP), U.S. Food and Drug Administration (FDA), Laurel, MD 20708 USA

**Keywords:** Microarray, RNA-seq, Toxicogenomics, Concentration response, BMC modeling, In vitro, Cannabinoid, Cannabichromene, Cannabinol

## Abstract

**Background:**

Transcriptomic benchmark concentration (BMC) modeling provides quantitative toxicogenomic information that is increasingly being used in regulatory risk assessment of data poor chemicals. Over the past decade, RNA sequencing (RNA-seq) is gradually replacing microarray as the major platform for transcriptomic applications due to its higher precision, wider dynamic range, and capability of detecting novel transcripts. However, it is unclear whether RNA-seq offers substantial advantages over microarray for concentration response transcriptomic studies.

**Results:**

We provide an updated comparison between microarray and RNA-seq using two cannabinoids, cannabichromene (CBC) and cannabinol (CBN), as case studies. The two platforms revealed similar overall gene expression patterns with regard to concentration for both CBC and CBN. However, in spite of the many varieties of non-coding RNA transcripts and larger numbers of differentially expressed genes (DEGs) with wider dynamic ranges identified by RNA-seq, the two platforms displayed equivalent performance in identifying functions and pathways impacted by compound exposure through gene set enrichment analysis (GSEA). Furthermore, transcriptomic point of departure (tPoD) values derived by the two platforms through BMC modeling were on the same levels for both CBC and CBN.

**Conclusions:**

Considering the relatively low cost, smaller data size, and better availability of software and public databases for data analysis and interpretation, microarray is still a viable method of choice for traditional transcriptomic applications such as mechanistic pathway identification and concentration response modeling.

**Supplementary Information:**

The online version contains supplementary material available at 10.1186/s12864-025-11548-3.

## Background

Modern toxicology calls for the use of new approach methodologies (NAMs) to address the 3Rs (Replacement, Reduction and Refinement) and to effectively generate more human-relevant data for the growing number of chemicals in the consumer markets that urgently need information for risk assessment and regulatory decision-making [1, 2]. A wide variety of methods have been developed as NAMs, including quantitative structure–activity relationship (QSAR) predictions, high-throughput screening (HTS) bioassays, omics applications, in vitro cell culture models and organoids, microphysiological systems (MPS), machine learning (ML) models and artificial intelligence (AI) [1]. Among these NAMs, transcriptomics provides a powerful high-throughput tool for exploring the genome-wide biological perturbations resulted from chemical exposures [3]. Combining transcriptomics with benchmark concentration (BMC) modeling provides quantitative toxicogenomic information that is increasingly used in regulatory risk assessment for chemicals with limited data [4].

Whole genome microarrays have been the primary platform for use in transcriptomics in the early years for over a decade [5]. Microarrays use hybridization-based approach to profile transcriptome-wide gene expression by measuring fluorescence intensity of predefined transcripts, and have the merits of relatively simple sample preparation, low per sample cost, and well-established methodologies of data processing and analysis [6]. However, microarrays have the disadvantages of limited dynamic range, high background noise, and nonspecific binding. Next generation RNA sequencing (RNA-seq) emerged in the mid 2000s as an alternative technology for transcriptomic profiling. RNA-seq is based on counting of reads that can be reliably aligned to a reference sequence. Therefore, RNA-seq virtually has no limit to the dynamic range of signal detection. More importantly, RNA-seq can identify transcripts not usually detectable by microarrays, including splice variants and non-coding transcripts such as microRNA (miRNA), long non-coding RNA (lncRNA), and pseudogenes. With the advancement of the technology and reduced cost, RNA-seq has gradually become the mainstream platform for transcriptomic studies in recent years [7].

Several studies have compared RNA-seq and microarray platforms for toxicogenomic applications [8–10]. These studies were limited to gene expression profiling after in vivo exposure to chemicals at a single dose. The comparisons were mainly focused on overall gene expression patterns, differentially expressed genes (DEGs) and associated functions or pathways. Dose or concentration response modeling of transcriptomic data has gained increasing importance over the past two decades, and has been recently proposed to exploit such transcriptomic data to refine and replace the current apical endpoint-based regulatory toxicity testing paradigm [11]. However, comparison on the performance of RNA-seq *vs*. microarray in the context of dose or concentration response modeling has been lacking. The only comparative dose response study in the literature between the two platforms was made more than 10 years ago [12], when both the RNA-seq technology and its data analysis algorithms were outdated from present point-of-view.

In the current study, we provide an updated comparison between microarray and RNA-seq with the most up-to-date technologies for both platforms. We conducted an RNA-seq experiment on cannabichromene (CBC) and cannabinol (CBN) using the same samples from a previous concentration response transcriptomics study using microarrays [13]. The results of the study showed that CBN was the most potent while CBC the least potent among the four cannabinoids tested. In recent years, many consumer products containing cannabinoids appear on the market [14, 15]. However, little is known about their safety or potential toxicity. Transcriptomic concentration response studies can yield quantitative toxicogenomic information that will contribute to risk assessment and regulatory decision-making of these data poor compounds. We analyzed the RNA-seq data using protocols widely accepted by the research community and compared the results to those obtained from the microarray study. Despite some degree of discordance between the two platforms found during data analysis, very similar final results, *i.e.* impacted functional pathways and transcriptomic point of departure (tPoD) values, were obtained by the two platforms.

## Methods

### Cell culture

Commercial iPSC-derived hepatocytes (iCell Hepatocytes 2.0) were obtained from FUJIFILM Cellular Dynamics (Madison, WI). Cells were cultured following the manufacturer’s protocol. Briefly, cells were thawed and seeded onto 24-well cell culture plates coated with rat tail collagen type I at a cell density of 3 × 10^5^ cells/cm^2^, using plating medium containing RPMI 1640 medium, B27 supplement, oncostatin M (20 ng/ml), dexamethasone (0.1 μM), gentamicin (25 μg/ml), and the proprietary iCell Hepatocytes 2.0 Medium Supplement. The plating medium was replenished daily for the first 4 days after seeding. On the fifth day, the plating medium was replaced by maintenance medium, which had the same composition as the plating medium but without oncostatin M. The maintenance medium was changed bidiurnally. Cells were ready for use between days 5–8 after seeding.

### Cannabinoid exposure

Purified CBC and CBN in DMSO were provided by the National Center for Natural Products Research at the University of Mississippi (University, MS). A stock solution of 40 mM for each cannabinoid was prepared in DMSO as described previously [16] and stored at −80 °C until use. Cannabinoid exposure was carried out on day 6 of cell culture. Cells cultured in 24-well plates were exposed to varying concentrations of cannabinoids in triplicate. Immediately before exposure, the stock solution was further diluted in DMSO to 200× of the nominal cannabinoid concentrations. Dosing solutions were prepared by diluting the 200× solutions in maintenance medium to the final concentrations, maintaining a DMSO concentration of 0.5% (v/v) across all treatments. Cells of the vehicle control groups were treated with maintenance medium containing 0.5% DMSO only. The exposure was conducted at 37 °C, 5% CO_2_ for 24 h.

### RNA sample preparation

Cells were lysed in RLT buffer (Qiagen, Valencia, CA) supplemented with 1% (v/v) β-mercaptoethanol at the end of the exposure and the lysates were stored at − 80 °C until RNA extraction. The lysates were thawed on ice and homogenized using QIAshredder (Qiagen). Total RNA was purified from the cell lysates using EZ1 Advanced XL automated RNA purification instrument (Qiagen) with the EZ1 RNA Cell Mini Kit (Qiagen), and instructions from the manufacturer were followed. An on-column DNase digestion step was included to remove contaminating genomic DNA. Total RNA concentration and purity (260/280) were subsequently measured using a NanoDrop 2000 UV–vis spectrophotometer (NanoDrop Products, Wilmington, DE). RNA quality was further checked using the Agilent 2100 Bioanalyzer (Agilent, Santa Clara, CA) with the RNA 6000 Nano Reagent Kit (Agilent) to obtain the RNA integrity number (RIN).

### Microarray data generation

All reagents and instruments used in the microarray experiment were obtained from Affymetrix (Santa Clara, CA). Total RNA samples were processed using the GeneChip 3' IVT PLUS Reagent Kit and hybridized onto GeneChip PrimeView Human Gene Expression Arrays following protocols from the manufacturer. Briefly, single-stranded complementary DNA (cDNA) was generated from 100 ng total RNA using reverse transcriptase and a T7-linked oligo(dT) primer, which was then converted to double-stranded cDNA using DNA polymerase and RNase H. Subsequently, complementary RNA (cRNA) was synthesized through in vitro transcription (IVT) with biotinylated UTP and CTP, using T7 RNA polymerase as the enzyme and the second strand of the double-stranded cDNA as the template.

The biotin-labeled cRNA was then purified and a fraction of 12 µg was fragmented by Mg^2+^ at 94 °C. Fragmented cRNA was then hybridized onto the microarray chips in the GeneChip Hybridization Oven 645 at 45 °C for 16 h. After hybridization, the microarray chips were stained and washed on the GeneChip Fluidics Station 450. Finally, the chips were scanned using the GeneChip Scanner 3000 7G, and the scanned image (DAT) files were further preprocessed using Affymetrix GeneChip Command Console software (v4.0) to produce cell intensity (CEL) files. The CEL files were imported into the Affymetrix Transcriptome Analysis Console (TAC) software (v4.0). Arrays were quality-checked prior to further data processing and analysis, and potential outliers were removed. The robust multi-chip average (RMA) algorithm integrated in the software were used to summarize values of individual probes belonging to one probeset in the CEL files, which consists of three steps: 1) background adjustment; 2) quantile normalization; and 3) summarization. The normalized expression data for each probeset (on log_2_ scale) were used for further data analysis.

### RNA-seq data generation

Sequencing libraries were prepared using the Illumina Stranded mRNA Prep, Ligation kit (San Diego, CA) following protocols provided by the manufacturer. Briefly, messenger RNAs (mRNAs) with polyA tails were purified from 100 ng of total RNA of each sample using oligo(dT) magnetic beads then fragmented and denatured. First strand complementary DNA (cDNA) was synthesized by reverse transcription of the hexamer-primed RNA fragments. Then a second strand cDNA was synthesized to replace the RNA template and generate blunt-ended, double-stranded cDNA fragments. In place of deoxythymidine triphosphate (dTTP), deoxyuridine triphosphate (dUTP) was incorporated to quench the second strand during amplification and achieve strand specificity. An adenine (A) nucleotide was added to the 3ʹ ends of the blunt fragments to prevent them from ligating to each other during adapter ligation. A corresponding thymine (T) nucleotide on the 3ʹ end of the adapter provides a complementary overhang for ligating the adapter to the fragment. Pre-index anchors were ligated to the ends of the double-stranded cDNA fragments to prepare them for dual indexing, and a subsequent PCR amplification step added the index adapter sequences. The adapter-ligated fragments were purified using magnetic beads. In the next step, a 13-cycle PCR was run to selectively amplify the anchor-ligated DNA fragments and add indexes and primer sequences for cluster generation. The resulting product is a dual-indexed library, *i.e.*, DNA fragments with adapters at each end. Finally, the dual-indexed libraries were purified using magnetic beads.

The libraries were analyzed using the Agilent 2100 Bioanalyzer and DNA 1000 Kit for size distribution and further quantified by Qubit Flex Fluorometer using the Qubit dsDNA BR Assay Kit (Thermo Fisher Scientific; Waltham, MA). The library of each sample was diluted to 2 nM in resuspension buffer (RSB) and 10 µl of the diluted libraries from all the samples were pooled. The pooled library is further diluted to 750 pM in RSB with Tween and 20 µl was loaded onto a thawed P3 200-cycle cartridge. Next, paired-end RNA-seq cluster generation and sequencing by synthesis was performed on the Illumina NextSeq 2000 instrument, following protocols provided by the manufacturer. Depths of an average 37.7 million of paired 75 bp reads were generated for each sample. Reads were aligned to the human reference genome hg38-alt-aware using the DRAGEN BCL Convert v3.8.4 workflow, and gene-level counts were obtained for each sample which were subsequently normalized by DESeq2 median of ratios in Partek Flow (Partek; Chesterfield, MO).

### Functional analysis by gene set enrichment analysis

Gene set enrichment analysis (GSEA) was conducted using the GSEA software (v4.2.2) from the Broad Institute (Cambridge, MA). The genomewide expression data between the two phenotype classes (*i.e.*, treatment group versus control group) were analyzed for enrichment in the H collection (hallmark gene sets, v7.5.1) found in the Molecular Signature Database (MSigDB). All other parameters of the software were set at the default values except for the permutation type, for which gene_set was used instead of phenotype. For microarray, expression data at probeset level were used; for RNA-seq, normalized counts of annotated genes after filtering out genes having “0” count for all samples were used.

### BMC modeling

BMC modeling was conducted using BMDExpress v.3 [17]. Recommendations as outlined in the NTP Research Report on National Toxicology Program Approach to Genomic Dose–Response Modeling [18] were followed with minor modifications. The normalized expression data were imported into BMDExpress. For microarray, expression data at probeset level were used; for RNA-seq, normalized counts of annotated genes after filtering out genes having “0” count in any sample were used. The expression data were first prefiltered using the Williams Trend Test to select probesets/genes that exhibit significant monotonically increasing or monotonically decreasing concentration response; a total of 100 permutations with FC cutoff > 1.5 and unadjusted *p*-value cutoff < 0.05 were used for the selection. Selected probesets/genes were further modeled (curve fitting) using the EPA Benchmark Dose Software (BMDS) models, including linear, hill, power, exponential (exp 3 and exp 5), and polynomial (poly 2, poly 3 and poly 4), to identify potential concentration response relationships and best fit. The model with the lowest Akaike information criterion (AIC) was selected as the best fit model. Hill models were flagged if the “k” parameter was less than 1/3 of the lowest concentration, and the next best model that meets both the minimum AIC value and a goodness-of-fit *p*-value > 0.05 was selected. A benchmark response (BMR) factor of 1.349 × standard deviation (SD) of the control samples was employed for the modeling assuming constant variance, using the profile likelihood method for BMCU and BMCL (statistical upper and lower bounds of computed BMC) estimation.

For subsequent functional classification (category analysis), probesets mapping to more than one gene, having a BMC value greater than the highest concentration (20 or 40 µM) or less than tenfold below the lowest concentration (0.1 µM), with goodness-of-fit *p*-value < 0.1, or with non-convergent BMC, BMCL, BMCU values (BMC/BMCL > 20, BMCU/BMC > 20, or BMCU/BMCL > 40) were all excluded. When two or more probesets are associated with a single gene, they were merged based on their NCBI Entrez Gene identifiers, and their BMCs were averaged to obtain a single value corresponding to the Entrez ID, *i.e.*, gene-level benchmark concentration. For genes with potentially conflicting probesets, a minimum correlation cutoff of 0.5 was applied; and genes with probesets not matching the minimum correlation cutoff were identified in the output. The Entrez IDs were then matched to various functional classifications including Gene Ontology Biological Process (GO_BP), Reactome pathways, and BioPlanet pathways.

## Results

### Overview of the study design and compound exposure

In a previous study [13], we conducted a transcriptomic concentration response study on hemp extract and its four major constituent cannabinoids, CBC, cannabidiol (CBD), cannabigerol (CBG), and CBN. Through BMC analysis of the transcriptomic data obtained from Affymetrix microarray, we compared their potency and also derived a tPoD value for each of the compounds. In the present study, we chose two of the cannabinoids, CBC (the least potent one among the four) and CBN (the most potent one), and conducted a similar concentration response study by Illumina RNA-seq using the same total RNA samples of the previous study. Our goal was to evaluate the concordance between the two transcriptomic platforms and to see if, for transcriptomic concentration response studies, similar tPoD values could be derived at the end of data analysis regardless of the platform used. Human iPSC-derived hepatocytes were exposed in triplicates to CBC (0, 0.1, 0.5, 1.0, 5.0, 10, 20, and 40 µM) and CBN (0, 0.1, 0.5, 1.0, 5.0, 10, and 20 µM) for 24 h. The concentrations were selected based on previously obtained cytotoxicity data [13].

### Specification of the platforms

Major specifications of the two transcriptomic platforms are listed in Table 1. The Affymetrix GeneChip PrimeView Human Gene Expression Array uses in situ oligonucleotide hybridization to measure the expression of ~ 50,000 probe sets, which cover > 36,000 transcripts and variants, and represent ~ 20,000 Entrez genes (mostly protein-coding genes). The robust multi-chip analysis (RMA) algorithm, which is the default method used by the TAC software, was used for data normalization.

**Table 1:** Transcriptomics platforms used for comparison

	**Microarray**	**RNA-seq**
Platform	Affymetrix GeneChip PrimeView Human Gene Expression Array	Illumina NextSeq 2000, with P3 XLEAP-SBS Reagents
Technology	In situ oligonucleotide hybridization	Next generation sequencing by synthesis (SBS) chemistry
Gene content	49,372 probe sets representing 19,866 Entrez genes	227,462 Ensembl transcripts mapping to 60,609 Ensembl genes (25,317 Entrez genes)
Normalization	Robust multi-chip average (RMA)	DESeq2’s median of ratios
Transformation	Log_2_	Log_2_

RNA-seq was performed on the Illumina NextSeq 2000 system using P3 200-cycle XLEAP-SBS Reagents. Reads were mapped to the human reference genome hg38-alt-aware using the DRAGEN BCL Convert v3.8.4 workflow. Read counts of ~ 227,000 Ensembl transcripts were converted to counts of ~ 60,000 Ensembl genes, of which ~ 25,000 Entrez genes (including ~ 20,000 protein-coding genes, ~ 5,000 non-coding RNA transcripts, pseudogenes, etc.). The gene-level read counts were then normalized using DESeq2’s median of ratios.

### RNA-seq data overview

Over the 45 samples (24 for CBC and 21 for CBN), the average number of passing filter (PF) reads was 29.2 million, of which 21.9 million (75%) mapped to the hg38-alt-aware human reference genome. Among the 24 CBC samples, one replicate of the 0.1 µM treatment group had very a low number of mapped reads (219) and was excluded from data analysis. Of the remaining 23 CBC samples, the numbers of mapped reads were in the range of 10.5–21.3 million with an average of 16.5 million per sample (Supplementary Table 1). In comparison, the 21 CBN samples had much higher numbers of mapped reads, ranging 23.1–37.8 million and averaging 28.9 million per sample (Supplementary Table 2). Among the mapped 60,609 Ensembl genes, only 16,698 (27.6%) had read counts greater than 0 in all the 23 CBC samples, and of which 13,399 (22.1%) had read counts greater than 10 in all the samples. Similarly, 17,705 genes (29.2%) had read counts greater than 0 in all the 21 CBN samples, of which 14,159 (23.4%) had read counts greater than 10 in all the samples.

### Comparison of overall gene expression patterns between the two platforms

**Fig. 1:**
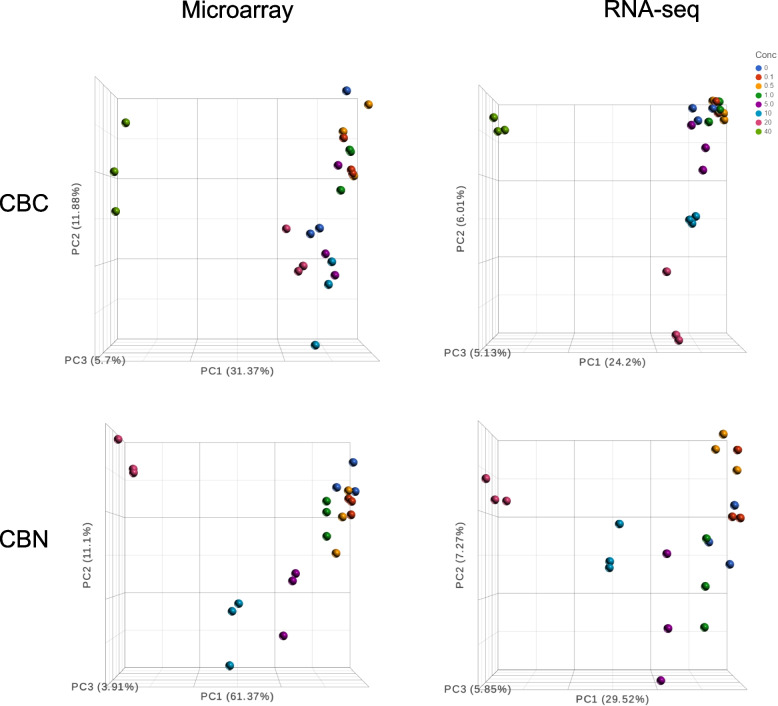
Principal component analysis (PCA) of the microarray and RNA-seq data for CBC and CBN. Samples (in triplicate, except for the 0.1 μM CBC group which was in duplicate) are color-coded by concentration as indicated in the top right corner of the graph. Percentages of contribution to variation for the first three principal components (PC1, PC2, and PC3) are indicated in the parentheses

The microarray and RNA-seq data were first compared by principal component analysis (PCA), which reduces the complexity of the transcriptomic data into multiple principal components (Fig. 1). Overall, PCA separated the concentration groups majorly along the first principal component (PC1) for both compounds in both platforms, but to a much lesser extent on the second and third principal components (PC2 and PC3). For CBC, PC1 accounted for 31% variance in the microarray dataset while only 24% in the RNA-seq dataset; moreover, the first three principal components (PC1, PC2, and PC3) together accounted for 49% variance in the microarray dataset versus 35% variance in the RNA-seq dataset. These numbers indicate that there were more noises (unknown factors) in the RNA-seq dataset, likely due to the large number of non-coding RNA transcripts which did not respond to concentration changes. This was even more evident in the case of CBN, where the variance accounted for by PC1 was 61% in microarray and only 30% in RNA-seq, and the variance accounted for by PC1, PC2, and PC3 together was 76% in microarray compared to 43% in RNA-seq.

**Fig. 2:**
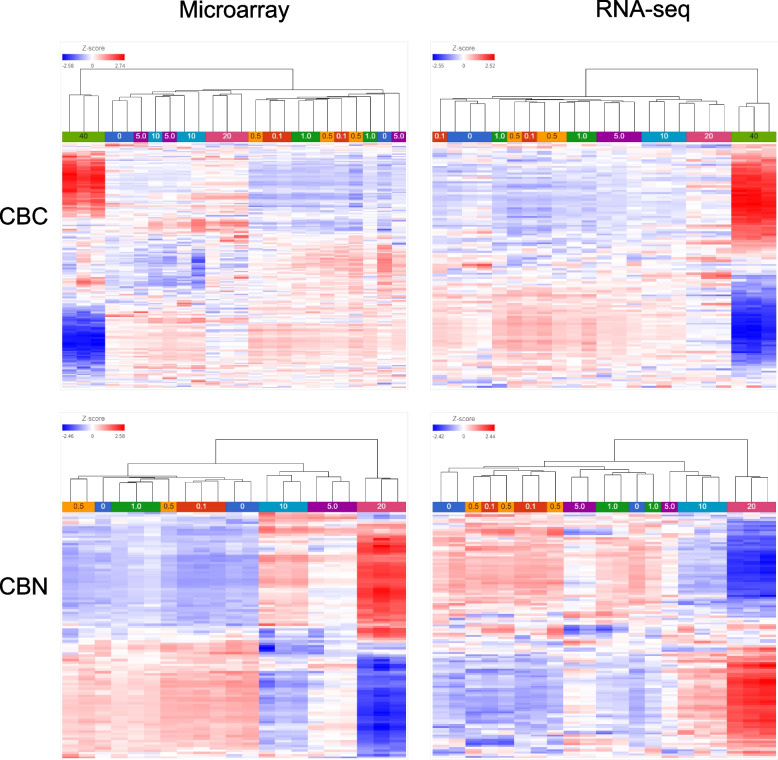
Hierarchical clustering analysis (HCA) of the microarray and RNA-seq data for CBC and CBN. Samples (in triplicate, except for the 0.1 μM CBC group which was in duplicate) were clustered as columns and color-coded by concentration as indicated below the dendrograms. The scheme of z-scores is shown at the top left corner of each map

The similarities in gene expression pattern among the samples were also explored by hierarchical clustering analysis (HCA) (Fig. 2). Overall, the sample dendrograms were generally similar between microarray and RNA-seq for both CBC and CBN. However, for CBC, sample clustering was better in RNA-seq than in microarray; conversely, sample clustering was better in microarray than in RNA-seq for CBN. For CBC, only samples of the two highest concentration groups (20 and 40 µM) clustered together respectively in the microarray data; samples at ≤ 10 µM did not cluster with their respective concentration groups. In the RNA-seq data, most samples cluster with their respective concentration groups except for the three lower concentrations (0.1, 0.5 and 1.0 µM). For CBN, except for two concentration groups (0 and 0.5 µM), all samples clustered with their respective concentration groups in the microarray data. Whereas in the RNA-seq data, only the two highest concentration groups (10 and 20 µM) clustered together.

### Gene expression concordance between microarray and RNA-seq

To form a common basis for comparison of gene expression between the two platforms, the expression data from microarray and RNA-seq were both mapped to Entrez gene identifiers (IDs). The 49,732 probesets on the Affymetrix PrimeView Human Gene Expression Array were first collapsed to genes, *i.e.*, expressions of probesets mapping to the same gene were combined and assigned a single Entrez gene ID. The expression of a total of 19,372 genes with unique Entrez IDs were generated from the microarray data as a result. The RNA-seq data were mapped to 26,739 Entrez genes in total. The two platforms overlap on 18,736 genes with unique Entrez IDs.

**Fig. 3:**
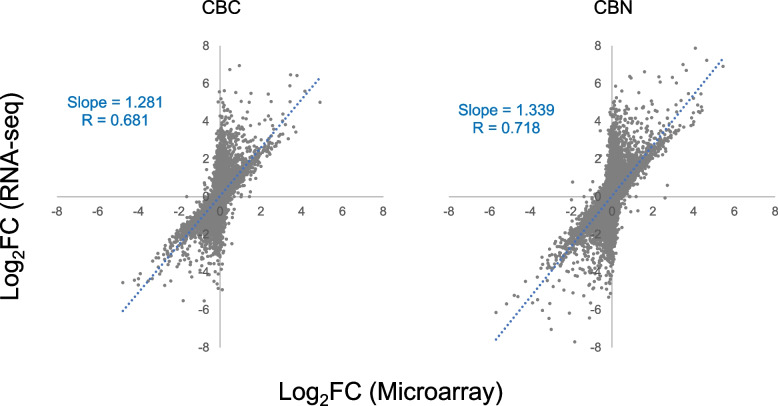
Scatter plots of fold change (FC) relative to the control on log_2_ scale (log_2_FC) of the 18,376 common genes between microarray and RNA-seq. Only the highest concentration group for CBC (40 μM) and CBN (20 μM) is depicted. The correlation coefficient and slope of the linear regression line (dotted blue) are included in each plot

A quantitative comparison of the relative raw expression profile of the 18,376 overlapped genes between the two platforms is shown in Fig. 3 for the highest concentration group of each compound. The fold change (FC) relative to the control on log_2_ scale (log_2_FC) for all the genes showed a general linear relationship between the platforms with Spearman rank correlation coefficients being 0.681 and 0.718 for CBC and CBN respectively, indicating a good concordance of the measured gene abundance changes between the two different gene expression profiling methods. The correlation slope of the linear regression line in both cases was higher than 1, indicating that RNA-seq generally had higher fold change estimates than microarray. In addition, there appeared to be a bifurcation in the scatter plot for both CBC and CBN. A smaller but significant portion of the genes scattered alongside the Y-axis instead of the regression line, suggesting some of these genes might be identified as DEGs by RNA-seq but not on microarray.

**Fig. 4:**
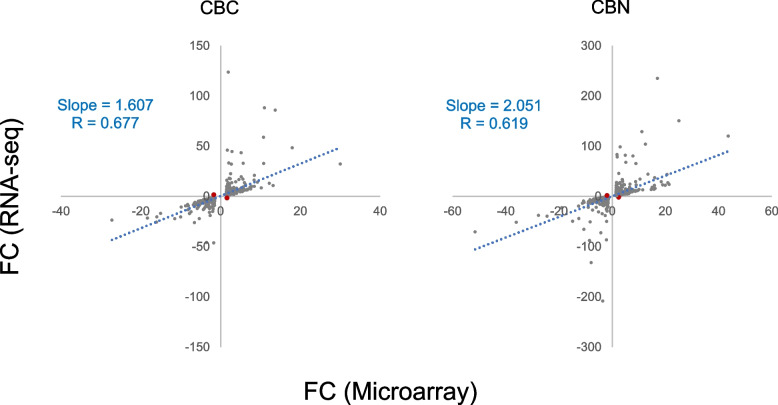
Scatter plots of fold change (FC) of the overlapped DEGs between microarray and RNA-seq. Only the highest concentration group for CBC (40 μM, 1726 overlapped DEGs) and CBN (20 μM, 2812 overlapped DEGs) is depicted. Genes with opposite directions of fold change between the two platforms are shown in red. The correlation coefficient and slope of the linear regression line (dotted blue) are included in each plot

Among the 18,736 overlapped genes between the two platforms, the numbers of those that were differentially expressed (|fold change (FC)|> 1.5, *p* < 0.05) by CBC or CBN exposure were compared between both platforms (Table 2). At each concentration, the microarray platform captured a much smaller number of DEGs (< 50%) compared to the RNA-seq platform for both compounds. Nevertheless, of the DEGs identified by microarray, in general over 80% were also captured by RNA-seq, except in a few cases where the exposure concentration was low and therefore only a small number of DEGs was identified (by microarray). The Spearman rank correlation coefficient for the overlapped DEGs of CBC at 40 µM was 0.677, and that of CBN at 20 µM was 0.619 (Fig. 4), revealing a good concordance between the two platforms. Moreover, both platforms showed the same directionality in expression changes for nearly all the shared DEGs, *i.e.*, only 2 out of the 1726 shared DEGs for CBC at 40 µM had opposite directions of fold change, and similarly, only 2 out of 2812 for CBN at 20 µM (Fig. 4), further demonstrating the concordance between the two platforms. However, as expected, RNA-seq showed much wider dynamic ranges of fold change for the overlapped DEGs compared to microarray at all concentrations for both compounds (Table 3, Fig. 4), which may explain, at least in part, the relatively low correlation coefficients described above.

**Table 2:** Comparison of DEGs (|FC|> 1.5, *p* < 0.05) between microarray and RNA-seq

Conc. (µM)	CBC			CBN		
	Microarray	RNA-seq	Overlap (%*)	Microarray	RNA-seq	Overlap (%*)
0.1	10	247	2 (20%)	0	177	---
0.5	27	361	22 (81%)	1	183	1 (100%)
1.0	10	235	6 (60%)	6	224	3 (50%)
5.0	2	167	2 (100%)	251	868	195 (78%)
10	20	296	17 (85%)	1074	2518	905 (84%)
20	130	577	112 (86%)	3050	5756	2812 (92%)
40	1925	3928	1726 (90%)	---	---	---

^*^percentage of overlapped genes between the two platforms relative to the number identified by microarray

--- data not available or could not be computed

**Table 3:** Comparison of dynamic range of fold change of overlapped DEGs between microarray and RNA-seq

Conc. (µM)	CBC		CBN	
	Microarray	RNA-seq	Microarray	RNA-seq
0.1	–1.92 – 1.55	–5.77 – 6.97	n/a – n/a	–4.80 – 11.66
0.5	–1.95 – 1.70	–5.34 – 5.58	n/a – 2.64	–6.89 – 25.14
1.0	–1.67 – n/a	–4.01 – 4.42	–1.67 – 6.37	–4.03 – 19.25
5.0	–1.64 – n/a	–3.86 – 11.20	–4.42 – 36.67	–11.16 – 88.49
10	–2.12 – 1.61	–4.12 – 4.34	–9.10 – 45.07	–302.92 – 113.95
20	–3.45 – 2.69	–6.81 – 7.65	–51.54 – 43.65	–302.92 – 234.95
40	–27.23 – 30.01	–46.30 – 123.71	---	---

--- data not available

n/a not applicable

### Comparison of functional analysis between microarray and RNA-seq

**Fig. 5:**
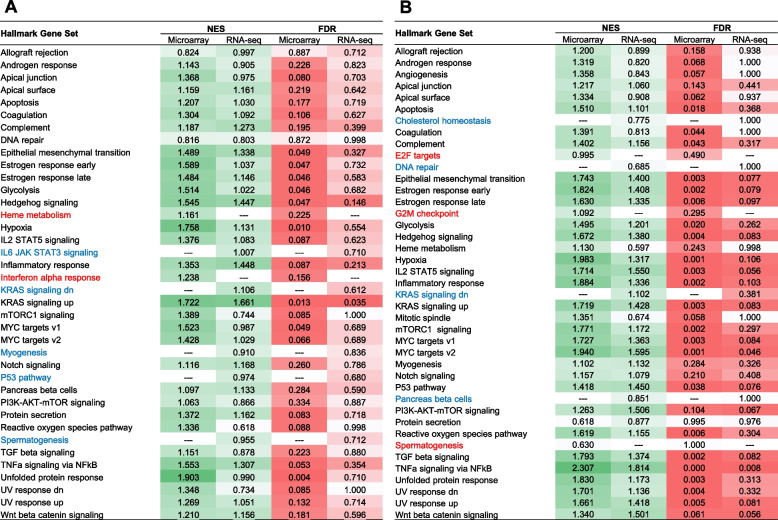
Gene set enrichment analysis (GSEA) for CBC (**A**) and CBN (**B**). The names of hallmark gene sets enriched by both platforms are shown in black. Those enriched only by microarray are shown in red and those only by RNA-seq in blue. The normalized enrichment score (NES) and false discovery rate (FDR) are listed for each enriched gene set and color-shaded based the NES and FDR values. Dark green indicates high NES score and light green low NES score, whereas dark red indicates low FDR value and light red high FDR value

One of the major goals of toxicogenomics studies is to elucidate the mechanism of action of toxicants through identifying biological functions and pathways impacted by their exposure. To understand if gene expression changes identified by microarray and RNA-seq lead to similar conclusions as to the biological effects of the toxicants, we performed GSEA on both datasets. The *hallmark* gene set collection from the Molecular Signatures Database (MSigDB), which contains 50 refined gene sets representing specific well-defined biological states or processes displaying coherent expression, was used for enrichment. Of the 50 hallmark gene sets, 33 were upregulated by exposure to 40 µM CBC as analyzed using the microarray data, and 36 were upregulated when analyzed using the RNA-seq data. Encouragingly, 31 enriched gene sets (82% out of 38 unique gene sets in total) overlapped between the two platforms (Fig. 5A). Only 2 gene sets were uniquely enriched in microarray and 5 in RNA-seq. Although RNA-seq identified a few more enriched gene sets, microarray generally had better enrichment scores and statistics for the overlapped gene sets between the two platforms.

For CBN at 20 µM, 37 of the 50 hallmark gene sets were enriched by microarray and 38 by RNA-seq. Among these, 34 overlapped between the two platforms, being 83% of the totally 41 unique gene sets (Fig. 5B). Similarly, gene sets enriched by microarray showed better enrichment scores and statistics in general, despite that RNA-seq identified a slightly higher number of enriched gene sets.

### Comparison of BMC modeling between microarray and RNA-seq

By default, the log_2_ transformed expression data of the 49,372 probesets on the microarray for both compounds were directly imported into BMDExpress for BMC analysis. For RNA-seq, the normalized, log_2_ transformed gene-level counts were first filtered to remove genes that had 0 value in any of the samples. The filtered expression data with 14,347 genes for CBC (Supplementary Table 3) and 14,869 genes for CBN (Supplementary Table 4) were uploaded for BMC analysis. The expression data from both microarray and RNA-seq were first prefiltered using two-sided William’s trend test to identify those for which there was a monotonically increasing or monotonically decreasing response to exposure concentration. The probesets/genes that passed the filtering were subjected to BMC modeling with a benchmark response of 1.349 × SD (equivalent to 10%). Model fitting was performed on each probeset/gene by fitting 8 different statistical models (including linear, hill, power, exp 3, exp 5, poly 2, poly 3, and poly 4) to the concentration response data assuming constant variance. The model that best described the data with the least AIC was selected. The BMC value derived from the best fit model of the probeset/gene (referred to as the best BMC) was taken as the BMC of the probeset/gene. All the modeled probesets/genes (for each compound) were further classified functionally by matching to the built-in defined categories (individual gene, GO_BP, Reactome pathways, and BioPlanet pathways).

**Fig. 6:**
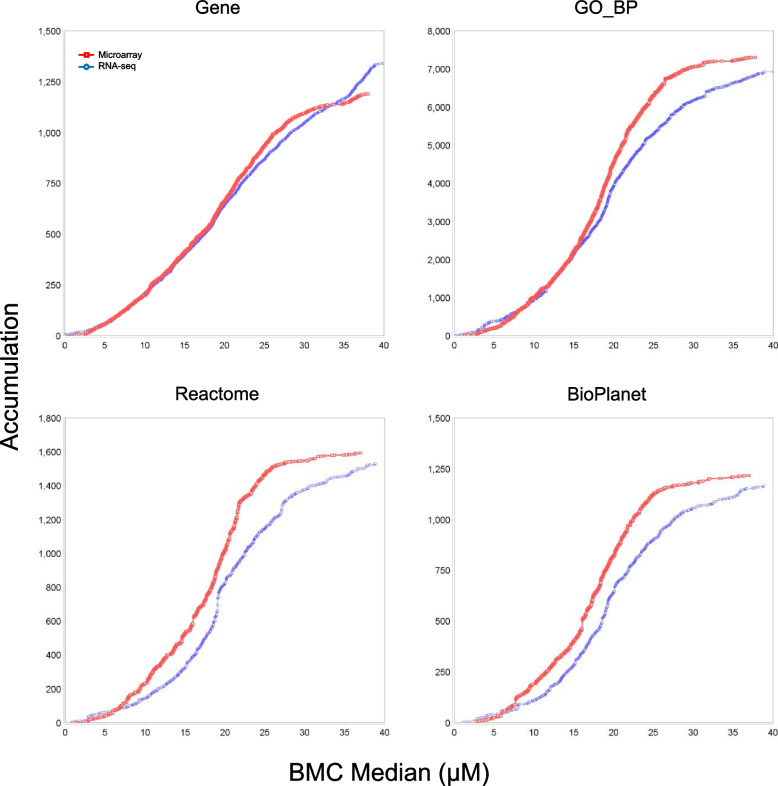
Comparison of median BMD accumulation of genes, GO_BP terms, Reactome pathways and BioPlanet pathways for CBC between microarray and RNA-seq

**Fig. 7:**
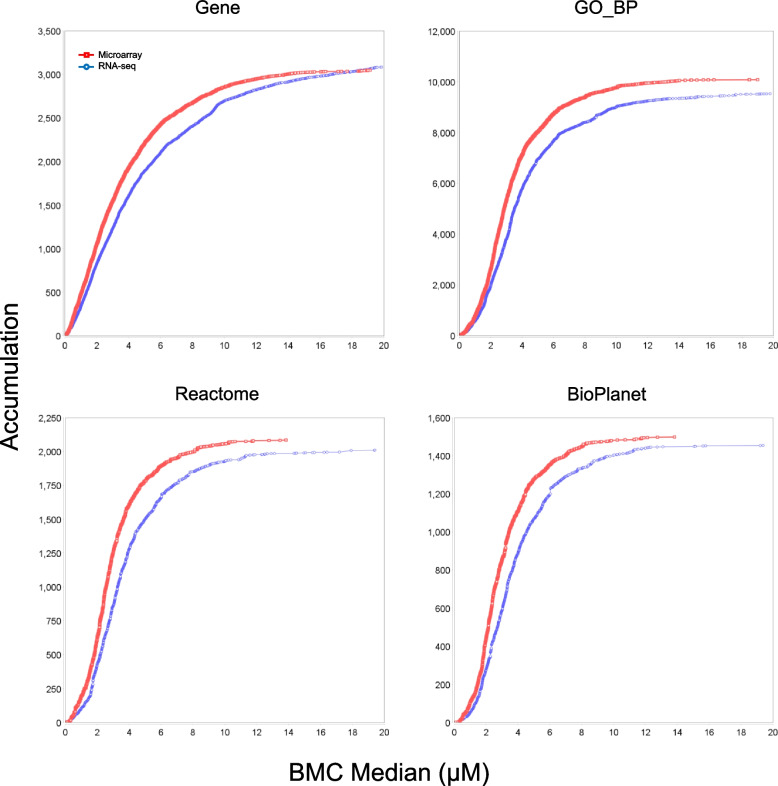
Comparison of median BMD accumulation of genes, GO_BP terms, Reactome pathways and BioPlanet pathways for CBN between microarray and RNA-seq

Comparison between the two platforms on concentration response characteristics were made using gene- and pathway-based BMC values. Table 4 shows an overview of the modeling results. Overall, for both compounds the two platforms agreed well in terms of both the numbers of resultant entries (genes, processes, and pathways) and the median BMC range, except that for GO_BP terms and Reactome and BioPlanet pathways, microarray had higher median BMC values for the low range limit for CBC and lower values for the high range limit for CBN. The general similarity between the platforms were also corroborated by the accumulation curves in Fig. 6 (for CBC) and Fig. 7 (for CBN), where the curves for all the functional classified groups displayed similar shapes between microarray and RNA-seq, albeit some slight differences in the curve slopes.

**Table 4:** Overview of BMC modeling results

	**CBC**				**CBN**			
	Microarray		RNA-seq		Microarray		RNA-seq	
	#^*^	Range^†^	#	Range	#	Range	#	Range
Input data^∞^	49,372	n/a	14,347	n/a	49,372	n/a	14,869	n/a
Prefiltered^‡^	4758	n/a	3941	n/a	7786	n/a	6053	n/a
Modeled^∫^	4748	0.012—39.699	3887	0.016—39.983	7780	0.010—19.271	6011	0.018—19.869
Gene^∑^	1190	0.012—38.020	1339	0.082—39.983	3048	0.017—19.145	3083	0.018—19.869
GO_BP^∑^	7295	1.328—37.795	6918	0.099—39.983	10,080	0.040—19.002	9527	0.018—19.801
Reactome^∑^	1591	1.328—36.994	1528	0.815—38.840	2084	0.040—13.838	2010	0.018—19.411
BioPlanet^∑^	1215	2.677—36.994	1164	1.162—38.840	1498	0.110 -13.838	1454	0.160—19.385

^*^ Number of entries (probesets, genes, GO_BP terms, and pathways)

^†^ Range of BMC in µM (best BMC for modeled probesets or genes, or median BMC for categorized genes, GO_BP terms or pathways) values

∞ For microarray the input expression data were at the probeset level while for RNA-seq at the gene level

^‡^ Number of entries (probesets or genes) after prefiltering with William’s trend test and subsequently used for modeling

∫ Modeled – modeled probesets or genes with a valid best BMC value (1/10 of the lowest concentration < BMC < highest concentration of the study)

∑ Resultant induvial genes, GO_BP terms and pathways (Reactome or BioPlanet) functionally categorized by BMDExpress

n/a, not applicable

**Fig. 8:**
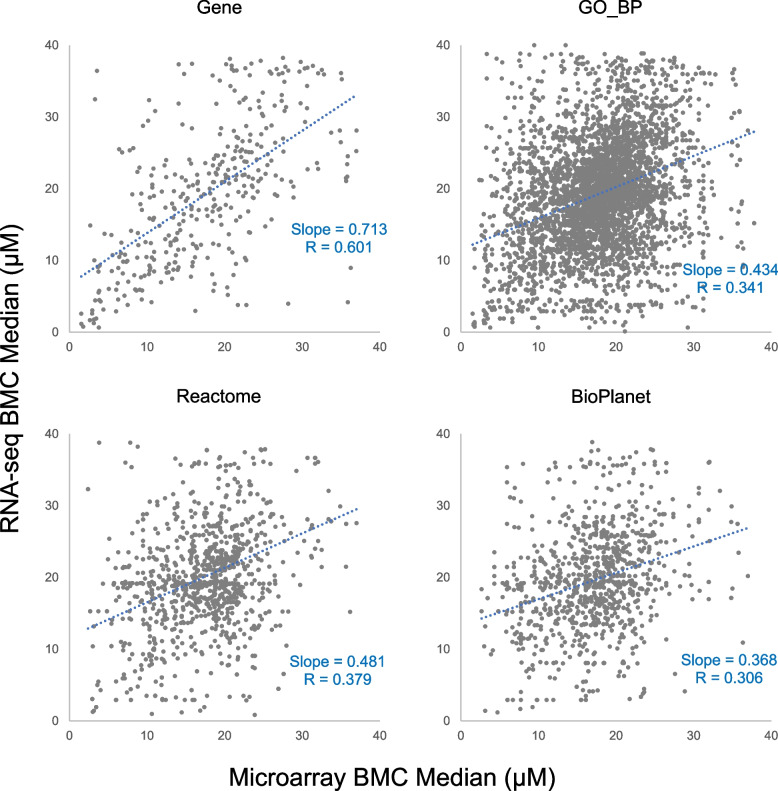
Scatter plots of median BMC of overlapped genes, GO_BP terms, Reactome pathways and BioPlanet pathways for CBC between microarray and RNA-seq. The correlation coefficient and slope of the linear regression line (dotted blue) are included in each plot

**Fig. 9:**
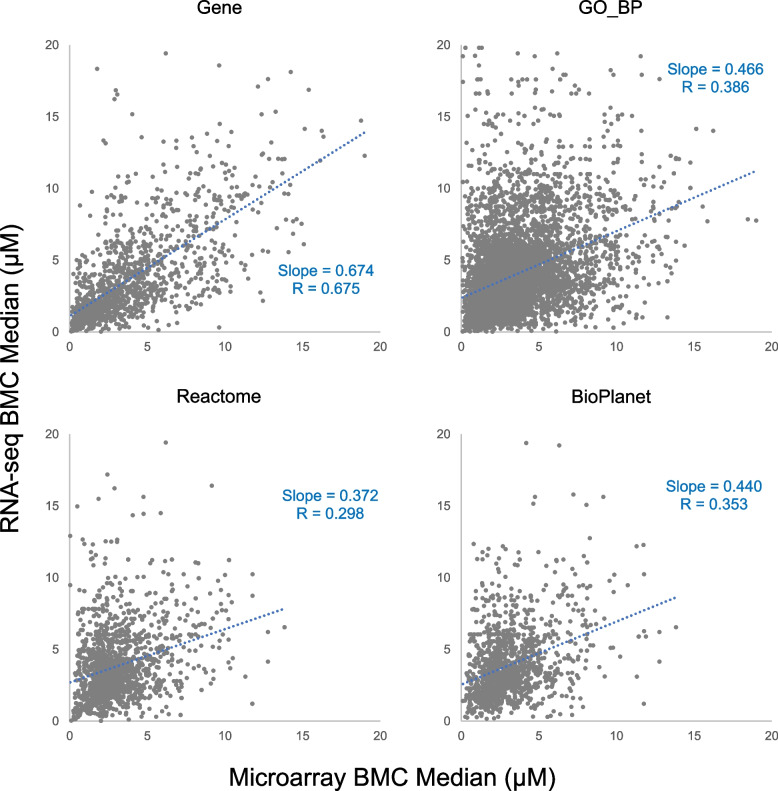
Scatter plots of median BMC of overlapped genes, GO_BP terms, Reactome pathways and BioPlanet pathways for CBN between microarray and RNA-seq. The correlation coefficient and slope of the linear regression line (dotted blue) are included in each plot

A closer look at the identity of the functional terms further revealed general agreement between the two platforms (Table 5). Despite low similarities at the gene level (overlap percentage being 16.5% and 27.0% for CBC and CBN respectively), similarities between the two platforms for the GO_BP terms and Reactome and BioPlanet pathways were fairly high, with overlap percentages being 64.3%, 71.5%, and 72.4% respectively for CBC, and 73.7%, 82.5%, and 87.8% respectively for CBN. Comparison of the overlapped genes showed general linear relationship with fairly good correlation coefficients of 0.601 for CBC (Fig. 8) and 0.675 for CBN (Fig. 9). Correlation coefficients for the GO_BP terms and Reactome and BioPlanet pathways were lower than genes, being 0.341, 0.379, and 0.306 respectively for CBC (Fig. 8), and 0.386, 0.298, and 0.353 respectively for CBN (Fig. 9). For both platforms, the BMC of functional terms fell largely in the range of 15 to 25 µM for CBC, while that for CBN fell below 5 µM, indicating the higher potency of CBN than CBC as previously described [13].

**Table 5:** Comparison of functionally classified terms between microarray and RNA-seq

		Microarray	RNA-seq	Overlap	Unique	%^a^
CBC	Gene	1190	1339	358	2171	16.5
	GO_BP	7295	6918	5564	8649	64.3
	Reactome	1591	1528	1300	1819	71.5
	BioPlanet	1215	1164	999	1380	72.4
CBN	Gene	3048	3083	1304	4827	27.0
	GO_BP	10,080	9527	8318	11,289	73.7
	Reactome	2084	2010	1851	2243	82.5
	BioPlanet	1498	1454	1380	1572	87.8

^a^Percentage of overlapped terms over the number of total unique terms

### Comparison of transcriptomic point of departure values between microarray and RNA-seq

To derive a tPoD, the median BMCL value of a functional term (GO_BP, Reactome pathway, or BioPlanet pathway) with the lowest median BMC value for each compound was identified. A compiled list of the functionally classified entries with the lowest median BMC from each function category is shown in Table 6 for CBC and Table 7 for CBN. For CBC, microarray identified GO_BP term 0008207: *C21-steroid hormone metabolic process* with a tPoD (or BMCL) value of 1.346 μM, whereas RNA-seq identified BioPlanet pathway 848: *benzo(a)pyrene metabolism* with a tPoD value of 0.917 μM. Similarly, a tPoD of 0.141 μM from GO term 0071395: *cellular response to jasmonic acid stimulus* was identified for CBN by microarray, and a tPoD of 0.099 μM from BioPlanet pathway 734: *nucleotide di- and triphosphate biosynthesis and interconversion* was identified by RNA-seq. For both compounds, the tPoD values derived from microarray and RNA-seq were on par with each other for the purpose of defining a threshold exposure value.

**Table 6:** Comparison of tPoD values derived for CBC from microarray and RNA-seq^*^

GO/Pathway ID	GO/Pathway Name	# All Genes	# Genes	Percentage	BMC Median	BMCL Median	BMCU Median
***Microarray***							
GO:1901661 (5)	Quinone metabolic process	38	3	7.9	1.777	1.346	2.379
GO:0007586 (3)	Digestion	34	3	8.8	1.777	1.346	2.379
*GO:0008207 (5)* ^†^	*C21-steroid hormone metabolic process*	*26*	*3*	*11.5*	*1.777*	***1.346***	*2.379*
R-HSA-156590	Glutathione conjugation	36	3	8.3	3.148	2.359	4.615
R-HSA-9818027	NFE2L2 regulating antioxidant/detoxification enzymes	17	3	17.7	3.148	2.359	4.615
1552	Glutathione conjugation	25	3	12.0	3.148	2.359	4.615
1739	Keap1-Nrf2 pathway	13	3	23.1	3.148	2.359	4.615
***RNA-seq***							
GO:0007586 (3)	Digestion	35	3	8.6	1.162	0.917	1.546
GO:0016137 (6)	Glycoside metabolic process	21	3	14.3	1.162	0.917	1.546
GO:0042448 (6)	Progesterone metabolic process	13	3	23.1	1.162	0.917	1.546
R-HSA-193368	Synthesis of bile acids and bile salts via 7alpha-hydroxycholesterol	24	3	12.5	1.162	0.917	1.546
*848*^†^	*Benzo(a)pyrene metabolism*	*9*	*3*	*33.3*	*1.162*	***0.917***	*1.546*

^*^Entry (-ies) with the lowest BMC median value in each category (GO_BP, Reactome, and BioPlanet) is (are) included. In many instances, multiple entries with equal BMC median values are included for each category. GO_BP ID starts with "GO:" followed by a number; Reactome ID starts with "R-HAS-" followed by a number; BioPlanet ID only contains a number. The numbers in the parentheses following a GO_BP ID indicate the GO level

^†^For multiple entries with equal lowest BMC median values, the one with the highest percentage (of genes) was selected as the tPoD defining biological process or pathway (highlighted in italic), and its BMCL median value is defined as the tPoD value (highlighted in bold)

**Table 7:** Comparison of tPoD values derived for CBN from microarray and RNA-seq^*^

GO/Pathway ID	GO/Pathway Name	# All Genes	# Genes	Percentage	BMC Median	BMCL Median	BMCU Median
***Microarray***							
GO:0042448 (6)	Progesterone metabolic process	13	3	23.1	0.223	0.141	0.369
GO:1902644 (5)	Tertiary alcohol metabolic process	15	5	33.3	0.223	0.141	0.369
GO:0030638 (4)	Polyketide metabolic process	10	5	50.0	0.223	0.141	0.369
GO:0030647 (7)	Aminoglycoside antibiotic metabolic process	10	5	50.0	0.223	0.141	0.369
GO:0044598 (4)	Doxorubicin metabolic process	10	5	50.0	0.223	0.141	0.369
GO:0044597 (5)	Daunorubicin metabolic process	9	5	55.6	0.223	0.141	0.369
GO:0009753 (5)	Response to jasmonic acid	4	3	75.0	0.223	0.141	0.369
* GO:0071395 (6)* ^†^	*Cellular response to jasmonic acid stimulus*	*4*	*3*	*75.0*	*0.223*	***0.141***	*0.369*
R-HSA-5336415	Uptake and function of diphtheria toxin	6	4	66.7	0.310	0.185	0.594
172	Acetylation and deacetylation of RelA in the nucleus	16	3	18.8	0.342	0.216	0.588
1598	NF-kappaB activation through FADD/RIP-1 pathway	12	3	25.0	0.342	0.216	0.588
***RNA-seq***							
GO:0032094 (4)	Response to food	24	4	16.7	0.342	0.153	0.931
R-HSA-9818027	NFE2L2 regulating antioxidant/detoxification enzymes	17	8	47.1	0.282	0.167	0.513
* 734*^†^	*Nucleotide di- and triphosphate biosynthesis and interconversion*	*18*	*3*	*16.7*	*0.160*	***0.099***	*0.281*

## Discussion

Microarray dominated in the early years of transcriptomics research [5]. Since the advent of next-generation sequencing (NGS) technology more than a decade ago, there have been constant comparisons between microarray and RNA-seq for various purposes including several for toxicogenomic applications [8–10]. These studies focused on gene expression patterns, DEGs, and associated functions or pathways. Overall, these studies indicate a reasonable concordance between the two platforms. Most published comparisons between microarray and RNA-seq have not evaluated dose or concentration response in gene expression, the only comparative dose response study was made more than 10 years ago [12], when the RNA-seq technology and its data analysis algorithms were still at their adolescence. Therefore, an updated comparison is needed now that both technologies have reached their maturity.

Affymetrix microarray and Illumina RNA-seq are two platforms that have gained popularity for transcriptomics studies. In the present study, we set out to provide an updated comparison between the two platforms each with the most up-to-date technologies. Concentration response transcriptomic data of two cannabinoids, CBC and CBN, were collected by the two platforms and compared. We first explored overall gene expression pattern of the data in relation to concentration using PCA (Fig. 1) and HCA (Fig. 2). Next, expression profile of the 18,376 common genes between the two platforms (Fig. 3) as well as the numbers (Table 2), dynamic ranges (Table 3) and directionalities (Fig. 4) of the DEGs were determined. Subsequently, we functionally analyzed the transcriptomic data by GSEA using the hallmark gene sets. Lastly, BMC modeling of the concentration response data was conducted and tPoD values were derived for the two compounds. Overall, consistent with previous reports [8–10, 12, 19], general concordance has been observed between the two platforms despite certain degrees of discrepancy in some specifics.

As seen in the results, comparisons between microarray and RNA-seq in many cases were not straightforward. First, the two platforms are fundamentally different in terms of data format. Microarray measures gene expression indirectly as fluorescence intensity resulted from hybridization with anti-sense probe sequences, whereas RNA-seq measures RNA transcript counts as a direct measurement of gene expression. Second, the two platforms use different annotations. The design of the probes on the PrimeView microarray was based on gene annotations from several sources including UniGene, RefSeq, NCBI genome, UCSC, Ensembl, GenBank, and Entrez; the mapping of the RNA transcripts was entirely based on the current human reference genome assembly, Genome Reference Consortium Human Build 38 (GRCh38). Impact of gene annotation choices has been discussed previously [20–22]. Third and more importantly, there was a huge cross-platform difference in the number of genes detected. On the microarray, there were 49,372 probe sets representing 19,866 Entrez genes; in comparison, RNA-seq detected 227,462 Ensembl transcripts mapping to 60,609 Ensembl genes, among which 25,317 were Entrez genes. Of the 60,609 Ensembl genes, only 18,736 overlapped with those on the microarray and of which most were protein-coding genes. The rest were non-coding RNA transcripts including miRNAs, lncRNAs, pseudogenes, and miscellaneous other RNA transcripts (miscRNAs). Since the current study focused on the application of BMC modeling which deals only with protein-coding genes, expression data on non-coding RNA transcripts were not analyzed. Nevertheless, it should be pointed out that expression of some non-coding RNAs such as miRNAs and lncRNAs are impacted by chemical exposure and therefore can be potentially used as toxicity biomarker and in some cases may offer additional insights into the mechanism of action of the toxicants [23, 24].

Consistent with previous studies [8–10], RNA-seq detected larger numbers of DEGs and also showed wider dynamic ranges of gene expression measurement (Tables 2 and 3). At lower concentrations, in particular, the numbers of DEGs detected by microarray were significantly smaller than those observed by RNA-seq for both CBC and CBN (Table 2). In the case of CBN, the DEGs detected by microarray at concentrations ≤ 1.0 µM might be considered negligible. These results suggest a lower sensitivity of the microarray platform compared with RNA-seq, *i.e.*, microarray is less capable of detecting low-expression genes or subtle changes in gene expression levels. This agrees with previous studies [25, 26] and may explain, at least in part, the fluctuations observed in the numbers of DEGs detected at low concentrations of CBC (Table 2). Nevertheless, the disadvantage of microarray in detection sensitivity did not seem to have obviously effect on its performance in functional analysis and BMC modeling (see discussions that follow).

A major goal of transcriptomics studies is through gene expression changes to predict the biological functions and pathways impacted by chemical exposure. It has been shown that RNA-seq reveals more significantly altered biological functions or pathways than microarray as a result of higher numbers of DEGs detected [10]. In our study, however, approximately equal numbers of functional gene sets were enriched from both platforms despite that greater numbers and wider dynamic ranges of DEGs were detected by RNA-seq. One likely explanation is that GSEA does not use lists of DEGs as input data but rather the whole transcriptome expression data [27, 28]. In addition, statistical methods used for DEG analysis are much different from those employed by GSEA. RMA, for microarray data processing, uses a linear model to fit the expression values which allows for comparisons between different conditions, and a moderated *t*-test to calculate DEGs [29]; DESeq2, in RNA-seq data analysis, uses negative binomial models to fit the count data and the Wald test for statistical hypothesis testing [30]. In contrast, the approach used by GSEA is radically different. GSEA primarily uses the Kolmogorov–Smirnov test as its underlying statistical method, which is a non-parametric test used to compare the distribution of a gene set within a ranked list of genes against a uniform distribution, allowing for the identification of enriched gene sets based on their ranking position rather than just their differential expression values alone [31]. Another possible explanation is that many of the DEGs identified by RNA-seq but not by microarray were actually non-coding RNAs (such as miRNAs and lncRNAs), which do not contribute to the enrichment of gene sets in GSEA.

Apart from the transcriptomic platforms, many other factors may affect the final results of BMC modeling, such as the bioinformatics workflow and various parameters used by the software programs in the workflow [32]. To make a fair and straightforward comparison between microarray and RNA-seq, we selected the most typical workflow for each platform and used default values for all the parameters used by the software programs in the workflow. Using default values worked well in most cases but may not be ideal for some parameters. For instance, the Williams Trend Test for prefiltering concentration response genes in BMDExpress performs 100 permutations by default to generate a null distribution of the test statistic. An increased number of 10,000 permutations has been recommended to generate more precise *p*-values and improve statistical power of the test [18, 33]. However, we found the increased permutations only had marginal effect on the numbers of genes passing the test and had no effect on the final tPoD values derived (data not shown). Method for data normalization is another important factor that may affect on the modeling results. In the study by Black et al. [12], the authors conducted an extensive comparison of different normalization methods, especially for RNA-seq. This was necessary as at the time of the study, RNA-seq normalization methods were still being developed, and it was then unclear which method was the best in providing robust normalization of technical variability across the relatively large dynamic range of count data while still maintaining biological fidelity. In the current study, no comparison was made on the various normalization methods for either platform. Instead, we chose the most widely used and best performer for each platform – RMA for microarray and DESeq2’s median of ratios for RNA-seq. The RMA normalization method was developed over two decades ago [34]. It has been widely accepted by the research community and implemented as the default normalization method in the Affymetrix TAC software. DESeq2’s median of ratios has also matured over a decade [35], and outperformed many other RNA-seq normalization methods for gene count comparisons between samples and for differential expression analysis [36]. Some previous studies found that the selection of normalization method did not have a strong influence on the final tPoD values [37, 38].

One of the major goals of a concentration response transcriptomic study is to derive a tPoD value for the chemical studied. The tPoD value could be regarded as the concentration below which no aggregated biological effect will occur. Through PBPK modeling, a reference dose (RfD) could be calculated from the tPoD as the safe exposure threshold for the chemical. Derivation of tPoD was based on previous studies demonstrating a good concordance between benchmark dose (BMD) values for phenotypical apical effects from traditional in vivo toxicity studies and transcriptomic BMDs of the most sensitive biological process, pathway, or gene set [39–41]. In our earlier study using microarray, we derived tPoD values for four cannabinoids (CBD, CBC, CBG, and CBN) [13]. Encouragingly, the tPoD for CBD, 0.106 μM, translates into an acceptable daily intake (ADI) of approximately 0.49 mg/kg-bw through physiologically based pharmacokinetic (PBPK) modeling, which is very close to an ADI value of 0.43 mg/kg-bw for CBD established previously based on sufficiently robust and reliable data identified from published human clinical trials and guideline-compliant toxicity studies in animal models (Henderson et al. 2023b). The concordance between the two ADI values derived by different approaches (in vitro transcriptomic endpoints *vs*. apical endpoints from clinical and animal studies) for CBD suggests the potential suitability of using tPoD to derive ADI for regulatory applications and adds confidence to the tPoD values derived for the other cannabinoids (CBC, CBG, and CBN). In the current study, the tPoD value derived from the RNA-seq data for CBC, 0.917 μM, was on the same level with that defined by microarray (1.346 μM) for the purpose of regulatory decision making. The same also held true for CBN (0.099 *vs*. 0.141 μM). Moreover, some functionally classified entries with the lowest median BMC in different categories overlapped between microarray and RNA-seq (Tables 6 and 7), further suggesting similar performance of the two platforms in defining tPoD through concentration response studies.

## Conclusions

The current study compared microarray and RNA-seq for concentration response study of two cannabinoids, CBC and CBN. Both platforms revealed similar overall gene expression patterns with regard to concentration. Although RNA-seq identified many varieties of non-coding RNA transcripts and larger numbers of DEGs with wider dynamic ranges, the two platforms performed equivalently in discovering functional gene sets (or pathways) impacted by compound exposure. Moreover, the two platforms derived similar tPoD values for both compounds through BMC modeling. Considering the relatively low cost, smaller data size (hence ease of data analysis and management), as well as better availability of software and public databases, microarray is still a viable method of choice for traditional transcriptomic studies where only the expression of protein-coding genes is concerned, such as mechanistic study through pathway identification and tPoD derivation through concentration response modeling.

## Supplementary Information

Supplementary Material 1: Table S1. Quantification of mapped reads for RNA-seq of CBC-exposed samples.

Supplementary Material 2: Table S2. Quantification of mapped reads for RNA-seq of CBN-exposed samples.

Supplementary Material 3: Table S3. Filtered RNA-seq expression data for CBC-exposed samples (14,347 genes) uploaded for BMC modeling.

Supplementary Material 4: Table S4. Filtered RNA-seq expression data for CBN-exposed samples (14,869 genes) uploaded for BMC modeling.

## Data Availability

The datasets generated and/or analyzed during the current study are available in the Gene Expression Omnibus (GEO; http://www.ncbi.nlm.nih.gov/geo/) repository of the National Center for Biotechnology Information with accession numbers GSE291936 (microarray, CBC), GSE291937 (microarray, CBN), GSE292355 (RNA-seq, CBC) and GSE292356 (RNA-seq, CBN). Other data are available from the corresponding author on reasonable request.
